# Treatment of Advanced-Stage Large Cell Neuroendocrine Cancer (LCNEC) of the Lung: A Tale of Two Diseases

**DOI:** 10.3389/fonc.2021.667468

**Published:** 2021-06-11

**Authors:** Tahani Atieh, Chao H. Huang

**Affiliations:** ^1^ Division of Medical Oncology, Department of Medicine, University of Kansas Medical Center, Kansas City, KS, United States; ^2^ Subpecialty Medicine, Kansas City VA Medical Center, Kansas City, MO, United States

**Keywords:** LCNEC, large cell neuroendocrine carcinoma, molecular profile, treatment, lung cancer, classification

## Abstract

LCNEC of the lung comprises a small proportion of pulmonary malignancies. Traditionally, they have been classified based on histologic and immunohistochemistry characteristics with features of small cell and non-small cell lung cancer. The treatment outcome of advanced-stage LCNEC of the lung is poor with response rates ranging from 34 to 46% with platinum doublets, median progression-free survival (mPFS) ranging between 4.4 and 5.8 m, and median overall survival (mOS) ranging from 8 to 12.6 m. The optimal treatment strategy for LCNEC is debated given limited data and different outcomes based on chemotherapy type reported in the available literature. Recently, genomic profiling with Next Generation Sequencing (NGS) has been able to sub-classify LCNEC as SCLC-like or NSCLC-like. Treatment based on this sub-classification has improved outcomes by using SCLC and NSCLC regimens based on their genomic profile in retrospective analysis. Future studies in LCNEC of the lung should incorporate this new molecular sub-classification as stratification and possibly include SCLC-like LCNEC into SCLC studies and NSCLC-like into NSCLC studies.

## Introduction

LCNEC of the lung comprises about 2 to 3.5% of all lung cancers. It is more frequent in males, with a median age of 65 years, and smokers (1). Travis et al. first described the LCNEC type of lung cancer in 1991 characterized by large cells with abundant cytoplasm, necrotic areas, high mitotic rate, and neuroendocrine features (2).

In 2004, The World Health Organization (WHO) classified LCNEC as a variant of large cell carcinomas. The 2015 update now has LCNEC in a group of neuroendocrine neoplasms with SLCL, typical carcinoids (TC), and atypical carcinoids (3). Karlson et al. showed through gene expression profiling that the WHO 2015 guidelines translated into a better transcriptional subgrouping of large cell carcinomas compared to the 2004 guidelines, but their analysis included a limited number of LCNEC and stressed the need for further molecular studies to tailor treatment (4).

## Pathology

The pathology of LCNEC is characterized by large cell size, abundant necrosis, low nuclear/cytoplasm ratio, neuroendocrine differentiation pattern associated with organoid nests, trabecular, rosette, and palisade formation. It also has a granular chromatin pattern, clear or atypical nucleoli. It has neuroendocrine markers such as chromogranin, neuron-specific enolase, synaptophysin, and somatostatin (3, 5). Pulmonary LCNEC shares pathologic features with atypical carcinoid in terms of growth pattern with LCNEC having more necrosis (6). The mitotic rate is usually high between 11 or more per 10 high power fields. A high mitotic rate helps differentiate between atypical carcinoid and small cell lung cancer (7). There could be features of non-small cell squamous or adenocarcinoma differentiation which is classified as mixed LCNEC. Because of these mixed features, it is not uncommon for mixed LCNEC to be diagnosed as poorly differentiated NSCLC or misclassified as atypical carcinoid or small cell lung cancer.

## Treatment of Advance Stage LCNEC

The treatment results of advanced-stage LCNEN have been controversial with retrospective studies showing conflicting results with SCLC regimens compared with NSCLC regimens. Please see **Table 1**. Sun et al. showed that the response rate was 73% using an SCLC regimen of cisplatin with etoposide compared with a response of 50% with NSCLC regimens (8). This was not the case in the retrospective review by Fujiwara et al. in which cisplatin with irinotecan had a lower response of 55% compared with a response rate of 71% with paclitaxel-based chemotherapy (9). A retrospective analysis by Naidoo et al. of 49 patients with stage IV LCNEC showed an ORR of 37% with first-line platinum/etoposide while no patients receiving non-platinum-based regimens had a response (10). The use of second-line therapy with Amrubicin by Yoshida et al. showed a low response rate of 27.7% and mPFS of 3.1 and mOS of 5.1 m (11). Immune checkpoint inhibitor (CKI) has been used to treat LCNEC, showing a response rate of 33%, mPFS of 4.2 m, and mOS of 11.8 months (12). These retrospective studies are limited by a small sample size and limited mutational analysis.

**Table 1:** Summary of treatments in LCNEC.

Study	Line of therapy	Regimen	ORR	mPFS	mOS
GFPC 0302 study Prospective phase II	Frontline	Cisplatin + Etoposide	34%	5 m	8 m
					
Niho et al. Japan. Prospective phase II	Frontline	Cisplatin + Irinotecan	46.7%	5.8 m	12.6 m
					
Christopoulos et al. German Prospective phase II	Frontline	Everolimus with paclitaxel and carboplatin	45%	4.4 m	9.9 m
					
Fujiwara et al. Retrospective review	Frontline	Cisplatin + Irinotecan Paclitaxel-based	55.6% 71%	4.1 m	10.3 m
					
Sun et al. Retrospective review	Frontline	Platinum + Etoposide/ Platinum + Taxane/gemcitabine/pemetrexed/vinorelbine/EGFR TKI	73% 50%	6.1 m 4.9 m	16.5 m 9.2 m
					
Yoshida et al. Retrospective review	Second line	Amrubicin	27.7%	3.1 m	5.1 m
					
Sherman et al. Retrospective review	10% 1st line 80% 2nd line 10% beyond 2nd line	Checkpoint inhibitor	33%	4.2 m	11.8 m

Prospective phase II trials showed response rates in the range of 34 to 46%, with platinum doublets and mPFS between 4.4 and 5.8 m, and mOS ranging from 8 to 12.6 m (13, 14). The addition of Everolimus to paclitaxel and carboplatin in a German study did increase response or improvement in mPFS and mOS (15).

## Molecular Classification and Outcome

The availability of sequencing technology has allowed further insights into the genomic make-up of LCNEC. This genomic characterization has implications in predicting chemotherapy outcome as two distinct molecular profiles have arisen: a molecular profile that behaves and responds like SCLC, and another profile that behaves and responds like NSCLC. The indication that LCNEC has genomic similarities with SCLC has been documented in early studies of immunochemistry and genomic expression comparing LCNEC and SCLC. These studies showed commonality with multiple genes and signal transduction pathways. The most common are TP53 and RB1 mutations, with TP53 mutation occurring in 75 to 95% of SCLC and 71 to 95% of LCNEC. The RB1 mutation occurs in 41 to 91% of SCLC and slightly lower at 26 to 38% of LCNEC (16).

RAS pathway alteration is also common in both disease states. An analysis of 15 LCNEC cases showed that it had an immunochemical expression of p53, point mutation of p53, c-RAF-1, and K-ras-2 indicating genetic similarity with SCLC (17). The PI3K/AKT/mTOR pathway alterations are commonly present in SCLC and LCNEC. A study from Japan performed genomic profile of SCLC and LCNEC and found similar genomic changes in the PI3K/AKT/mTOR pathway: PI3KCA (3%), PTEN (4%), AKT2 (4%) RICTOR (5%), mTOR (1%) and alterations in EGFR (1%), ERBB2 (4%) and FGFR1 (5%) in LCNEC compared with 4, 6, 2, 6, 1, 1 and 3% in SCLC respectively. Certain mutations seem to be more frequent in LCNEC, including LAMA1 (10%), PCLO (6%) and MEGF8 (5%) compared to SCLC. Copy numbers of ERBB2 and SETBP1 occurred in 4% of LCNEC (17). Other mutations were more frequent in SCLC, including the MYC amplification which occurs in 18–30% of SCLC compared to 23% of LCNEC. LCNEC can also have a genomic profile similar to NSCLC with alterations in SKT-11, KRAS, and KEAP1 (16). These studies showed that LCNEC is in reality two types of lung cancer; SCLC-subtype and NSCLC-subtype based on genomic profile.

A recent paper proposed a new molecular classification of LCNEC: type I with STK11/KEAP1 alterations, but with an NE phenotype, high expression of ASCL1 and DLL3, and downregulation of NOTCH pathway, as in the SCLC classical subtype; type II characterized by RB1 alterations, but a predominant non-NE phenotype (with low expression of chromogranin A and synaptophysin), high levels of REST and NOTCH, and immune cell response activation more responsive to NSCLC type therapy (18). A study in 94 cases of stage IV LCNEC showed DLL3 expression in 74% of cases. It was also positive in 76% of pRB negative, and 67% of pRB positive patients; 79% of RB1 mutated, and 70% of RB1 wildtype patients; 100% of STK11 mutated, 91% in KEAP1 mutated, and in 100% of cases with TP53 wildtype tumors. DLL3 expression was also associated with expression of ASCL1 and at least two out of three neuroendocrine markers (19).

Rekhtman et al. performed NGS in pulmonary LCNEC and found altered TP53 (78%), RB1 (38%), STK11 (33%), KEAP1 (31%), and KRAS (22%) and segregated LCNEC into two main different molecular subtypes: SCLC-like with TP53/RB1 inactivation and MYCL amplification; NSCLC-like with retained TP53/RB1 functions, NOTCH mutations and also with STK11/KRAS/TTF1 mutations, similar to that of adenocarcinoma, or KEAP1 mutations or SOX2/FGFR1 amplification, as with squamous cell carcinoma. They also proposed a third carcinoid subtype that had inactivation of MEN1 mutations and low mutation burden like carcinoid (20).

**Figure 1:**
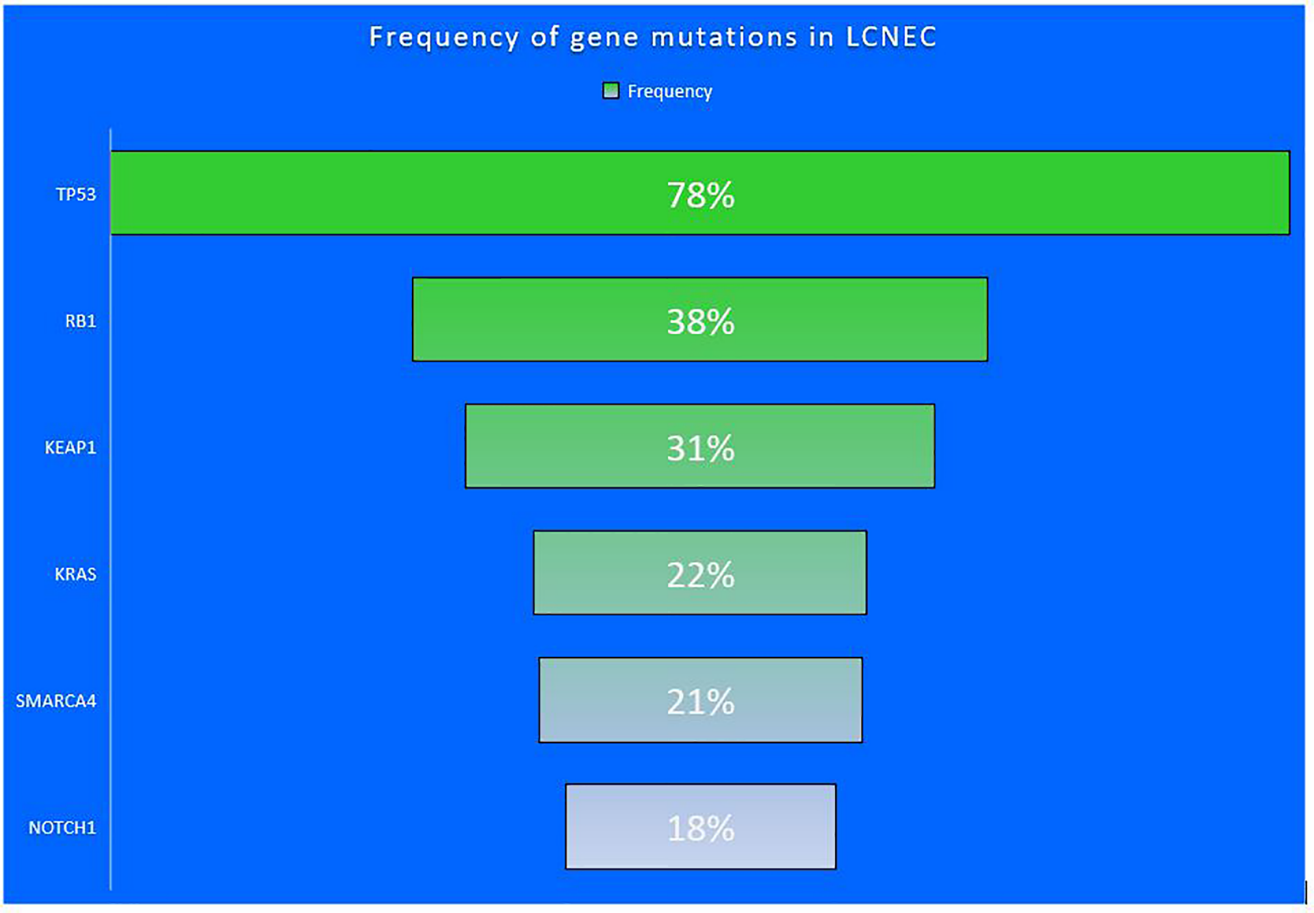
Frequency of gene mutations seen in LCNEC.

Similarly, George et al. used whole-exome/genome sequencing showed that LCNEC is composed of two mutually exclusive subgroups: “type I LCNECs” with bi-allelic TP53 and STK11/KEAP1 alterations (37%), and “type II LCNECs” enriched for bi-allelic inactivation of TP53 and RB1 (42%). Type I LCNECs with high neuroendocrine expression and, similar to SCLC, a profile of ASCL1 high/DLL3 high/NOTCH low, and type II LCNECs with reduced expression of neuroendocrine genes and a pattern of ASCL1 low/DLL3 low/NOTCH high (21). Derks et al. performed next-generation sequencing (NGS) and determined the TP53, RB1, STK-11, KEAP-1, and IHC for RB1 and P16 in patients with LCNEC and correlated mutations with treatment and clinical outcomes. This study found the frequency of RB1 mutation and protein loss to be 47 and 72% respectively. Cases with LCNEC with RB wild type treated with gemcitabine with taxane had significantly longer overall survival compared with those treated with small cell platinum and etoposide (PE) regimen. This study indicated that LCNEC with wild type RB1 treated with gemcitabine and taxane had a better outcome than using PE (22). Another study profiled tumor DNA and circulating tumor DNA (ctDNA). Commonly altered genes in these LCNEC tumors included TP53 (75%), RB1 (32.1%), SMARCA4 (21.4%), NOTCH1 (17.9%), KEAP1 (17.9%) with few cases of KRAS, EGFR, and CDKN2A mutations and STK11 mutation or loss (see **Figure 1**). The study then classified LCNEC as SCLC-like if RB1 or TP53 were mutated or had copy number loss and as NSCLC-like LCNEC if patients did not have RB1 and TP53 co-alterations (23).

In a case series of 95 LCNEC samples, 16% expressed PD-L1 and 5% had a tumor proportion score (TPS) of ≥50%. Its expression was equal in RB1 mutated and RB1 wild-type tumors. None of the STK11 mutated tumors expressed PD-L1. PD-L1 expression was correlated with superior overall survival (OS), hazard ratio 0.55 [(95% Confidence Interval 0.31–0.96), p = 0.038] (24). These studies showed that LCNEC should have a molecular profile done to distinguish if it is NSCLC-like or SCLC-like so that we can tailor the chemotherapy regimen. The studies using NGS to determine the genomic profile of LCNEC separated them into SCLC-like or NSCLC-like were able to show a different outcome when the cases are selected and treated based on their SCLC-like or NSCLC-like classification.

While NGS is timely and costly, IHC staining for RB1 and TP53 protein is readily available with much lower cost and may be a substitute to NGS to potentially differentiate these two subtypes (25). In a retrospective analysis, Myoshi et al. found that most of the RB1-mutated samples (93%) were negative for RB staining, and there was mutual exclusivity of protein expression between RB and p16 (26).

In a study by Karlson et al. also tumor protein p53 gene (TP53) and RB1 mutations were found in 91 and 82% of LCNEC tumors, respectively, with RB1 mutations always concurrently with TP53 mutations, and 91% of cases showed absent RB1 protein expression. Further, RB1 mutations seem to happen in a mutually exclusive way with mutations in STK11, KEAP1, and the KRAS/NRAS/HRAS pathway genes (27).

**Figure 2:**
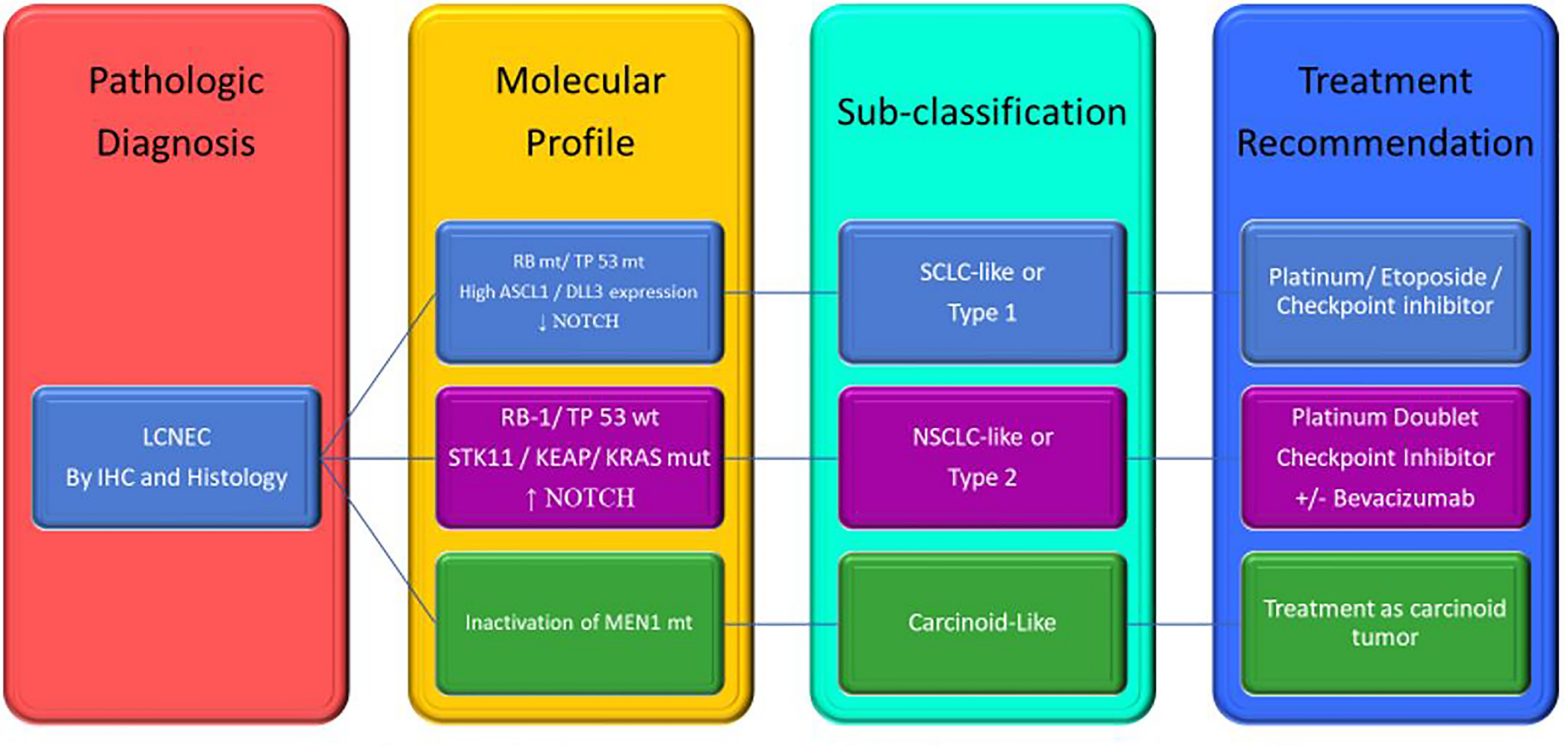
Proposed of LCNEC sub-classification based on genomic changes and treatment recommendation. LCNEC, large cell neuroendocrine cancer; IHC, immunochemistry; mt, mutant; wt, wild type; SCLC, small cell lung cancer; NSCLC, non-small cell lung cancer. Adapted from Rekhtman et al. (20).

Based on this information, it seems rational to start separating LCNEC based on genomic profile and classify them as SCLC-subtype, NSCLC-subtype, Carcinoid-subtype, and choose the regimen based on their genomic profile. See **Figure 2**.

## Conclusion

The nature of pulmonary LCNEC has been an ongoing enigma for the last several decades. LCNEC are diagnosed histologically with features of either small cell or non-small cell lung cancer. Early immunohistochemistry studies of pulmonary LCNEC also showed this dichotomy, demonstrating alterations of RB1 and TP53 common in SCLC with other samples lacking alterations of RB1 or TP53. Further molecular studies confirmed what was seen in the histologic and immunohistochemistry studies. They further confirmed the presence of dual molecular profiles of pulmonary LCNEC with an SCLC-subtype with mutations in RB1, TP53, MYCL amplification, and alterations in the PI3K/AKT/mTOR pathway; NSCLC-subtype with mutations in SKT-11, KEAP, and KRAS. A small subset of LCNEC has alterations of MEN-1 with histologic features of carcinoid. The emerging data is starting to define that pulmonary LCNEC is akin to Janus, the ancient god with two faces indicating the beginning and the end of the conflict, with two main subtypes: SCLC-subtype and NSCLC-subtype. This subclassification has treatment implications. The outcome of pulmonary LCNEC is worse than the typical NSCLC, likely because some of them have a natural history similar to SCLC. The overall treatment outcome of advanced LCNEC is generally poor with 5-year survival ranging from 15 to 25%. The treatment of advanced metastatic LCNEC generally consisted of an SCLC regimen of platinum and etoposide. Other trials have used regimens tailored toward NSCLC.

Recently, several papers proposed to use molecular profile testing to first sub-classify LCNEC into an SCLC-subtype or NSCLC-subtypes. The outcome of SCLC-subtype and NSCLC-subtype LCNEC seems to have a better outcome if they are treated based on the respective subtype of LCNEC as demonstrated by trials using this approach. This certainly makes clinical sense as the current treatment approach is to customize the treatment using an SCLC or NSCLC regimen. The use of molecular profile to make the treatment decision would make this a rational choice as opposed to an intuitive choice. The availability of next-generation sequencing technology should make the adoption of molecular sub-classification of LCNEC seemly in the current practice setting. We may not necessarily have to wait for confirmation of prospective trials using the approach of managing LCNEC based on molecular classification. Instead, we should focus our resources and research efforts on the identification of effective therapy for LCNEC. Future trials in lung cancer should include SCLC-subtype and NSCLC-subtype of LCNEC in respective disease state studies to answer important questions regarding the use of immunotherapy and specific targeted therapy so that we can move this field forward. This will expedite the approval of new treatment options and truly make an impact on the management of pulmonary LCNEC. 

## Author Contributions

TA and CHH: wrote, edited, reviewed, and finalized tables and figures. TA helped edit and review the current manuscript. All authors contributed to the article and approved the submitted version.

## Conflict of Interest

The institution where CH worked received research funding from the following companies to conduct clinical trials: Genentech/Roche, Bayer, Bristol-Mayers, Incyte, Sanofi, Astra Zeneca, Nektar, RGX, and Pfizer.

The remaining author declares that the research was conducted in the absence of any commercial or financial relationships that could be construed as a potential conflict of interest.
